# Supplementary material for: Asynchronous magnetic resonance elastography: Shear wave speed reconstruction using noise correlation of incoherent waves
Source: Magn Reson Med. 2022 Oct 27;89(3):990–1001. doi: 10.1002/mrm.29502 (PMC9792433; doi:10.1002/mrm.29502)
Supplement: Supplementary file 1 — DATA S1 MATLAB code used to generate the numerical simulations of Figure 1 [file MRM-89-990-s002.zip › k-Wave/helpfiles/maxND.html]

maxND :: Functions (k-Wave)


# maxND

Return the value and indices of the largest value in an N-D array.

## Syntax

```
max_val = maxND(mat)
[max_val, ind] = maxND(mat)
```

## Description

`maxND` returns the largest value in the N-D array given by `mat`. The indices of the largest value within the array can also be returned, where `ind = [ind_dim_1, ind_dim_2, ... , ind_dim_N]`. The indices can be used to directly address the largest value, i.e., `max_val = mat(ind)`.

## Inputs

|  |  |
| --- | --- |
| `mat` | input array |

## Outputs

|  |  |
| --- | --- |
| `max_val` | largest value in `mat` |
| `ind` | array of indices corresponding to the location of the largest value in `mat` |
